# Development and validation of predictive models for SIRS and severe hemorrhage following percutaneous nephrolithotomy: the role of hydronephrosis and inter-correlation

**DOI:** 10.1186/s12893-026-03721-6

**Published:** 2026-04-11

**Authors:** Wenlong Wan, Junyi Yang, Dongfeng Yuan, Yongqi Wang, Baokang Wang, Weisong Wu, Xiao Yu

**Affiliations:** 1 Department of Urology, Tongji Hospital of Tongji Medical College of Huazhong, University of Science and Technology, Wuhan, Hubei Province China; 2 Department of Urology, Union Hospital of Tongji Medical College of Huazhong, University of Science and Technology, Wuhan, Hubei Province China

**Keywords:** PCNL, Complications, SIRS, Hemorrhage, Predictive Model

## Abstract

**Objectiv:**

Postoperative systemic inflammatory response syndrome (SIRS) and severe hemorrhage are two major complications threatening patient safety after percutaneous nephrolithotomy (PCNL). This study aimed to investigate their risk factors, develop and validate predictive models, and analyze the impact of hydronephrosis severity on prognosis, as well as the potential association between these two complications.

**Methods:**

Clinical data from 708 patients with kidney stones who underwent PCNL were retrospectively analyzed. An additional 104 patients were selected as an external validation cohort. Independent risk factors for postoperative SIRS and severe hemorrhage were identified using univariate and multivariate logistic regression analyses, and clinical prediction models were constructed. Model performance was evaluated using the Hosmer-Lemeshow goodness-of-fit test, receiver operating characteristic (ROC) curve analysis, and decision curve analysis (DCA). Spearman’s correlation analysis was used to examine the correlation between SIRS and severe hemorrhage. Postoperative stone-free status was defined as residual fragments ≤ 4 mm, and severe hemorrhage was defined as the need for blood transfusion and/or interventional embolization.

**Results:**

Multivariate analysis showed that prolonged operative time (OR = 1.016), positive preoperative urine culture (OR = 3.651), and residual stones (OR = 2.680) were independent risk factors for postoperative SIRS. For postoperative severe hemorrhage, the independent factors were prolonged operative time (OR = 1.014), positive preoperative urine culture (OR = 2.763), and moderate hydronephrosis (OR = 0.113). The predictive models for SIRS and severe hemorrhage, constructed based on these variables, both demonstrated good discrimination and calibration. In the development cohort, the area under the ROC curve (AUC) for the SIRS model and the hemorrhage model were 0.761 (95% CI: 0.659–0.863) and 0.807 (95% CI: 0.719–0.895), respectively. In the external validation cohort, the corresponding AUC values were 0.773 (95% CI: 0.586–0.961) and 0.800 (95% CI: 0.618–0.982). Subgroup analysis revealed that patients with moderate hydronephrosis had the lowest risk of severe postoperative hemorrhage (0.9%) and the highest stone clearance rate (92.1%). Furthermore, a significant positive correlation was observed between the occurrence of postoperative SIRS and severe hemorrhage (*r* = 0.176, *P* < 0.001).

**Conclusion:**

This study successfully developed and validated predictive models for SIRS and severe hemorrhage after PCNL, demonstrating moderate-to-good clinical discriminatory ability. Moderate hydronephrosis may be a protective factor against hemorrhage after PCNL. The occurrence of postoperative SIRS and severe hemorrhage are not independent events; they may share interrelated pathophysiological mechanisms, warranting clinical attention and further investigation.

**Supplementary Information:**

The online version contains supplementary material available at 10.1186/s12893-026-03721-6.

## Introduction

Urolithiasis is one of the most common diseases in urology. Its high incidence and recurrence rates place a heavy burden on China’s healthcare system and significantly impact patients’ quality of life [1, 2]. Since its introduction in 1976, percutaneous nephrolithotomy has gradually replaced traditional open surgery for managing large or complex renal stones due to its advantages of minimally invasive access, fewer complications, and high stone-free rates.

Although PCNL is widely used due to its minimal trauma and reduced hemorrhage, its potential complications cannot be ignored, especially given the large volume of procedures performed. Common complications include hemorrhage, infection, injury to adjacent organs, and urine extravasation. Among these, infection and hemorrhage are the most critical, directly threatening patient safety and requiring particular attention in clinical practice [3, 4]. As the most life-threatening complications after PCNL, the mechanisms underlying SIRS and severe hemorrhage are complex and potentially interrelated. Therefore, identifying independent risk factors for these two complications and constructing clinical prediction models are of significant clinical importance for achieving early postoperative warning, enabling timely intervention, and improving patient outcomes.

Hydronephrosis is a common accompanying condition in patients with kidney stones. Its severity can affect renal parenchymal thickness, collecting system space, and intraoperative perfusion pressure, potentially influencing surgical difficulty, stone clearance rates, and postoperative hemorrhage and infectious complications [5]. Previous studies suggest that the degree of hydronephrosis may be associated with outcomes after PCNL; however, a unified and clear conclusion is currently lacking.

Systemic inflammatory response syndrome is an uncontrolled, excessive inflammatory response of the body to factors such as severe injury, infection, trauma, major surgery, or organ ischemia [6]. As a common complication after PCNL, SIRS is closely associated with poor patient outcomes, increasing the risk of mortality by 82%, with reported incidence rates ranging from 9.8% to 43% [7]. SIRS is often considered a precursor to sepsis; if not promptly identified and managed, it can progress to septic shock, which carries a higher mortality rate [8].

Postoperative hemorrhage is another common complication of PCNL. Minor hemorrhage may be asymptomatic and not life-threatening, but severe hemorrhage often requires active intervention and even surgical management [9, 10]. Failure to promptly recognize and correct hypovolemia can lead to life-threatening conditions such as shock [11].

The factors influencing PCNL complications are complex and diverse. Due to variations in data from included studies, regional differences in medical standards, and ethnic factors, no unified consensus has been reached in the academic community. Identifying the true determinants among numerous confounding variables remains a challenge in current research [12, 13]. This study aimed to construct and validate clinical prediction models for SIRS and severe hemorrhage after PCNL, with the goal of providing early warning in clinical practice, helping healthcare professionals increase vigilance, and enabling timely identification and intervention to reduce the incidence and mortality associated with these serious complications.

## Methods

### Population

This study was approved by the Ethics Committee of Tongji Hospital, Tongji Medical College, Huazhong University of Science and Technology. We retrospectively included 708 patients who underwent PCNL performed by our surgical team between January 2016 and January 2022. An external validation cohort consisted of 104 patients who underwent PCNL at our hospital between January 2023 and May 2023.

Inclusion criteria were: (1) underwent PCNL treatment; (2) had complete preoperative computed tomography imaging data to confirm urolithiasis; (3) had complete postoperative imaging data (plain abdominal film, ultrasound, or CT).

Exclusion criteria were: (1) patients with horseshoe kidney, solitary kidney, or post-kidney transplant; (2) patients with underlying diseases such as malignant tumors, hematological disorders, or immune system diseases; (3) patients with incomplete or missing preoperative urinary tract CT imaging data; (4) presence of uncontrolled systemic infection preoperatively (fever or white blood cell count > 10 × 10⁹/L); (5) age < 18 years.

### Data collection

Based on literature review and expert consultation, we retrospectively collected the following parameters: Demographic characteristics: sex, age, body mass index; Laboratory tests: preoperative serum creatinine, urine nitrite, urine white blood cell count, urine culture, blood glucose, prothrombin time, albumin, globulin, triglycerides, cholesterol, C-reactive protein, and albumin-to-globulin ratio; Surgery-related information: operative time; Postoperative conditions: need for blood transfusion and/or interventional embolization for hemostasis; body temperature, heart rate, respiratory rate, white blood cell count; Imaging data: preoperative stone size, stone location, degree of hydronephrosis, presence of residual stones postoperatively; Medical history: previous surgical history, history of anticoagulant medication use.

### Definitions

SIRS: The diagnosis of postoperative SIRS required meeting two or more of the following criteria: [1] heart rate > 90 beats/min; [2] body temperature > 38 °C or < 36 °C; [3] white blood cell count > 12 × 10⁹/L or < 4 × 10⁹/L, or immature neutrophil percentage > 10%; [4] respiratory rate > 20 breaths/min or arterial partial pressure of carbon dioxide < 32 mmHg.

Severe Postoperative Hemorrhage: Defined as the need for blood transfusion (Clavien-Dindo grade II) and/or interventional embolization for hemostasis (Clavien-Dindo grade IIIa) after surgery.

Residual stones were defined as the presence of residual fragments with a maximum diameter greater than 4 mm, confirmed by imaging studies such as plain abdominal film, ultrasound, or CT within three days postoperatively.

Hydronephrosis Grade: The degree of hydronephrosis was determined based on the basic morphology of the kidney, kidney size, cortical thickness, and separation of the renal sinus. The criteria were as follows: [1] No or mild hydronephrosis: essentially normal renal morphology, renal sinus separation < 2 cm; [2] Moderate hydronephrosis: dilatation of the major calyces, essentially normal morphology of the minor calyces, renal sinus separation between 2 and 3 cm; [3] Severe hydronephrosis: abnormal renal morphology, dilatation of the minor calyces, significant thinning of the renal cortex, renal sinus separation > 3 cm.

### Statistical analysis

Statistical analysis was performed using SPSS 25.0 software. Normality tests were conducted for continuous variables. Data following a normal distribution were expressed as mean ± standard deviation, and comparisons between groups were made using the independent samples t-test. Data not following a normal distribution were expressed as median (interquartile range), and comparisons between groups were made using the Mann-Whitney U test. Categorical variables were expressed as frequencies (percentages), and comparisons between groups were made using the Chi-square test or Fisher’s exact test. Univariate and multivariate logistic regression analyses were used to identify factors associated with postoperative SIRS and severe hemorrhage. Variables with *P* < 0.05 in the univariate analysis were included in the multivariate analysis. The variance inflation factor was used to assess multicollinearity, with VIF > 1.5 considered indicative of collinearity. The Hosmer-Lemeshow test was used to assess model calibration. Model performance was evaluated using the area under the ROC curve, calibration curves, and decision curve analysis. The Chi-square test and Spearman’s rank correlation analysis were used to examine the relationship between postoperative SIRS and severe hemorrhage. Common influencing factors for both complications were also analyzed using univariate and multivariate logistic regression. Variables used for model construction were imported into R software (version 4.3.2) and StataMP 17 to develop nomogram prediction models and generate ROC curves, calibration curves, and DCA curves. *P* < 0.05 was considered statistically significant.

### Surgical procedure

All patients underwent routine preoperative evaluation and received prophylactic antibiotics according to the HALF classification. Under general anesthesia, patients were initially placed in the lithotomy position. A 5 Fr ureteral catheter was inserted retrograde and connected to an irrigation system, and a 16 Fr urinary catheter was placed for drainage. The patient was then repositioned prone. Under ultrasound guidance, percutaneous puncture was performed through the 11th or 12th intercostal space. Successful access was confirmed by the free flow of urine. The tract was dilated to F18 ~ F24. Based on stone characteristics, fragmentation was achieved using a thulium laser and/or pneumatic lithotripter. The fragments were then irrigated and removed. Upon completion of the procedure, a nephrostomy tube and a double-J ureteral stent were placed and secured. Patients were monitored in the ward for 1–2 days postoperatively, with close observation of drainage, signs of infection, and hemorrhage.

## Results

The development cohort included 708 patients, of whom 457 (64.5%) were male, with a median age of 51 (39, 64) years. The postoperative incidence of SIRS was 5.9%, severe hemorrhage was 4.5%, and the concurrent occurrence of both was 1.1%.

Compared to the non-SIRS group, the SIRS group had higher rates of positive preoperative urine culture (41.2% vs. 11.7%), positive urine white blood cells (75.6% vs. 52.5%), and residual stones (31.0% vs. 12.9%); operative time was also longer [98 (72, 128) min vs. 69 (52, 90) min]. Compared to the non-severe hemorrhage group, the severe hemorrhage group had higher rates of positive preoperative urine culture (32.0% vs. 12.7%) and residual stones (40.6% vs. 12.7%), with a significantly different distribution of hydronephrosis: the proportion of moderate hydronephrosis was significantly lower (6.3% vs. 33.3%), while the proportions of no/mild hydronephrosis (43.8% vs. 30.3%) and severe hydronephrosis (50.0% vs. 36.4%) were higher. The median operative time was longer in the severe hemorrhage group [103 (68, 137) min vs. 69 (52, 90) min] (Supplementary Table 1).

In the external validation dataset of 104 patients, postoperative SIRS occurred in 8 patients, severe hemorrhage in 4 patients. Preoperative urine culture was positive in 20 patients and negative in 84. Stones were cleared in 75 patients, residual stones were present in 29. There were 28 patients with no/mild hydronephrosis, 49 with moderate hydronephrosis, and 27 with severe hydronephrosis. Operative time was 68 (52, 97) min (Supplementary Table 2).

Univariate logistic regression analysis with postoperative SIRS as the outcome identified seven variables with statistical significance (*P* < 0.05): stone diameter (*P* = 0.005, OR = 1.279, 95% CI: 1.078–1.514), operative time (*P* < 0.001), preoperative albumin-to-globulin ratio (*P* = 0.048), positive preoperative urine culture (*P* < 0.001), positive preoperative urine nitrite (*P* = 0.005), positive preoperative urine white blood cells (*P* = 0.005), and residual stones (*P* = 0.002) (Table 1). VIF analysis showed all VIF values were < 1.5, indicating no significant multicollinearity.

**Table 1:** Univariate Logistic Regression Analysis of Postoperative SIRS

	B	*P*	OR	95% CI	
				Lower	Upper
Age	-0.017	0.18	0.983	0.959	1.008
Stone Diameter	0.246	0.005	1.279	1.078	1.516
Preoperative Hb	-0.286	0.419	0.751	0.376	1.503
BMI	-0.592	0.082	0.553	0.284	1.079
Operative Time	0.016	< 0.001	1.016	1.009	1.024
Blood Glucose	0.029	0.763	1.029	0.854	1.241
Prothrombin Time	-0.079	0.736	0.924	0.585	1.461
Albumin	-0.017	0.685	0.983	0.907	1.066
Globulin	0.058	0.051	1.059	1	1.123
Triglycerides	-0.008	0.944	0.992	0.79	1.245
Cholesterol	-0.096	0.614	0.908	0.626	1.319
Serum Creatinine	0.001	0.521	1.001	0.998	1.005
C-reactive Protein	0.001	0.939	1.001	0.978	1.024
Albumin-to-Globulin Ratio	-1.111	0.048	0.329	0.109	0.989
Sex	0.227	0.484	1.255	0.664	2.372
Urine Culture	1.66	< 0.001	5.262	2.525	10.964
Single Stone	-0.89	0.147	0.411	0.123	1.369
Residual Stones	1.106	0.002	3.023	1.513	6.041
Urine Nitrite	1.47	0.005	4.347	1.543	12.25
Urine White Blood Cells	1.045	0.005	2.842	1.371	5.894
Hydronephrosis		0.664			
Hydronephrosis	-0.367	0.369	0.693	0.311	1.543
Hydronephrosis	-0.123	0.741	0.885	0.427	1.833
Surgical		0.951			
Surgical (1)	-18.513	0.998	0	0	
Surgical (2)	-0.155	0.752	0.856	0.328	2.239

Further multivariate analysis revealed that prolonged operative time (*P* = 0.005, OR = 1.016, 95% CI: 1.005–1.028), positive preoperative urine culture (*P* = 0.005, OR = 3.651, 95% CI: 1.490–8.948), and residual stones (*P* = 0.026, OR = 2.680, 95% CI: 1.124–6.391) were independent risk factors for postoperative SIRS (Table 2).

**Table 2:** Multivariate logistic regression analysis of postoperative SIRS

	B	*P*	OR	95% CI	
				Logwer	Upper
Operative Time	0.016	0.005	1.016	1.005	1.028
Urine Culture	1.295	0.005	3.651	1.49	8.948
Urine Nitrite	0.639	0.344	1.894	0.504	7.111
Stone Diameter	-0.017	0.89	0.984	0.776	1.246
Urine White Blood Cells	0.527	0.267	1.695	0.667	4.303
Residual Stones	0.986	0.026	2.68	1.124	6.391
Albumin-to-Globulin Ratio	-0.085	0.901	0.919	0.24	3.513

**Fig. 1:**
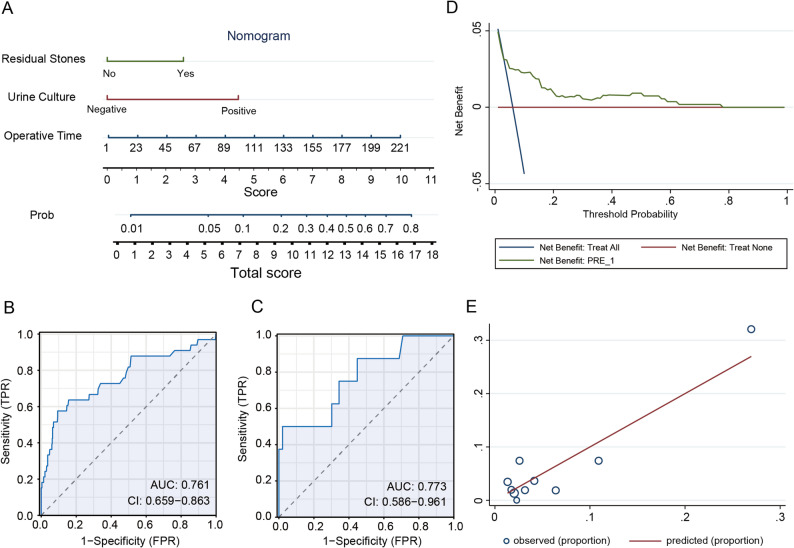
Illustrates the construction and validation of a predictive nomogram for postoperative SIRS risk following PCNL. The nomogram, which incorporates residual stones, urine nitrite, and operative time, is presented in Fig. 1**A**. The model demonstrated acceptable predictive performance, with an AUC of 0.761 (95% CI: 0.659–0.863) in the training set (Fig. 1**B**) and a comparable AUC of 0.773 (95% CI: 0.586–0.961) in the validation set (Fig. 1**C**). Decision curve analysis (Fig. 1**D**) revealed that the nomogram provided a positive net benefit across a range of threshold probabilities, indicating its clinical utility. Furthermore, the calibration curve (Fig. 1**E**) showed excellent agreement between the predicted probabilities and the actual observed incidence of SIRS, confirming the model's accuracy

The predictive model constructed based on these factors demonstrated good calibration (Hosmer-Lemeshow test, *P* = 0.548). The area under the ROC curve was 0.761 (95% CI: 0.659–0.863) in the development cohort and 0.773 (95% CI: 0.586–0.961) in the validation cohort. Calibration curves and decision curve analysis further confirmed the model’s performance (Fig. 1).

Univariate analysis with postoperative severe hemorrhage as the outcome showed that the following factors were statistically significant: operative time (*P* < 0.001), residual stones (*P* < 0.001), preoperative hemoglobin level (*P* = 0.007), positive preoperative urine culture (*P* = 0.009), and the degree of hydronephrosis (*P* = 0.023) (Table 3). VIF analysis showed all VIF values were < 1.5, indicating no significant multicollinearity.

**Table 3:** Multivariate logistic regression analysis of severe hemorrhage

	B	*P*	OR	95% CI	
				Lower	Upper
Age	0.025	0.089	1.025	0.996	1.055
Stone Diameter	0.093	0.447	1.097	0.864	1.393
Preoperative Hb	-0.987	0.007	0.373	0.181	0.767
BMI	-0.613	0.107	0.541	0.257	1.141
Operative Time	0.018	< 0.001	1.018	1.01	1.026
Blood Glucose	0.075	0.368	1.078	0.916	1.269
Prothrombin Time	0.325	0.154	1.383	0.885	2.162
Albumin	-0.065	0.151	0.937	0.857	1.024
Globulin	0.048	0.156	1.049	0.982	1.12
Triglycerides	-0.122	0.624	0.885	0.542	1.443
Residual Stones	1.546	< 0.001	4.694	2.238	9.847
Cholesterol	0.026	0.899	1.026	0.687	1.534
Serum Creatinine	0.001	0.639	1.001	0.997	1.005
C-reactive Protein	0.012	0.123	1.012	0.997	1.028
Albumin-to-Globulin Ratio	-1.102	0.08	0.332	0.097	1.142
Sex	0.093	0.804	1.097	0.527	2.283
Urine Culture	1.175	0.009	3.237	1.344	7.797
Single Stone	-1.111	0.134	0.329	0.077	1.409
Urine Nitrite	1.091	0.09	2.976	0.842	10.518
Urine White Blood Cells	0.527	0.166	1.695	0.804	3.571
Hydronephrosis		0.023			
Hydronephrosis	-2.039	0.007	0.13	0.029	0.58
Hydronephrosis	-0.049	0.897	0.952	0.454	1.998
Surgical		0.941			
Surgical (1)	0.259	0.731	1.296	0.295	5.696
Surgical (2)	-0.017	0.975	0.983	0.335	2.886

Multivariate analysis ultimately identified prolonged operative time (*P* = 0.007, OR = 1.014, 95% CI: 1.004–1.025), positive preoperative urine culture (*P* = 0.036, OR = 2.763, 95% CI: 1.067–7.158), and moderate hydronephrosis (*P* = 0.040, OR = 0.113, 95% CI: 0.014–0.910) as independent factors influencing postoperative severe hemorrhage (Table 4).

**Table 4:** Multivariate logistic regression analysis of severe hemorrhage

	B	*P*	OR	95% CI	
				Lower	Upper
Operative Time	0.014	0.007	1.014	1.004	1.025
Urine Culture	1.016	0.036	2.763	1.067	7.158
Residual Stones	0.821	0.099	2.273	0.858	6.022
Preoperative Hb	-0.653	0.156	0.521	0.211	1.283
Hydronephrosis		0.118			
Hydronephrosis	-2.18	0.04	0.113	0.014	0.91
Hydronephrosis	-0.09	0.847	0.914	0.367	2.277

**Fig. 2:**
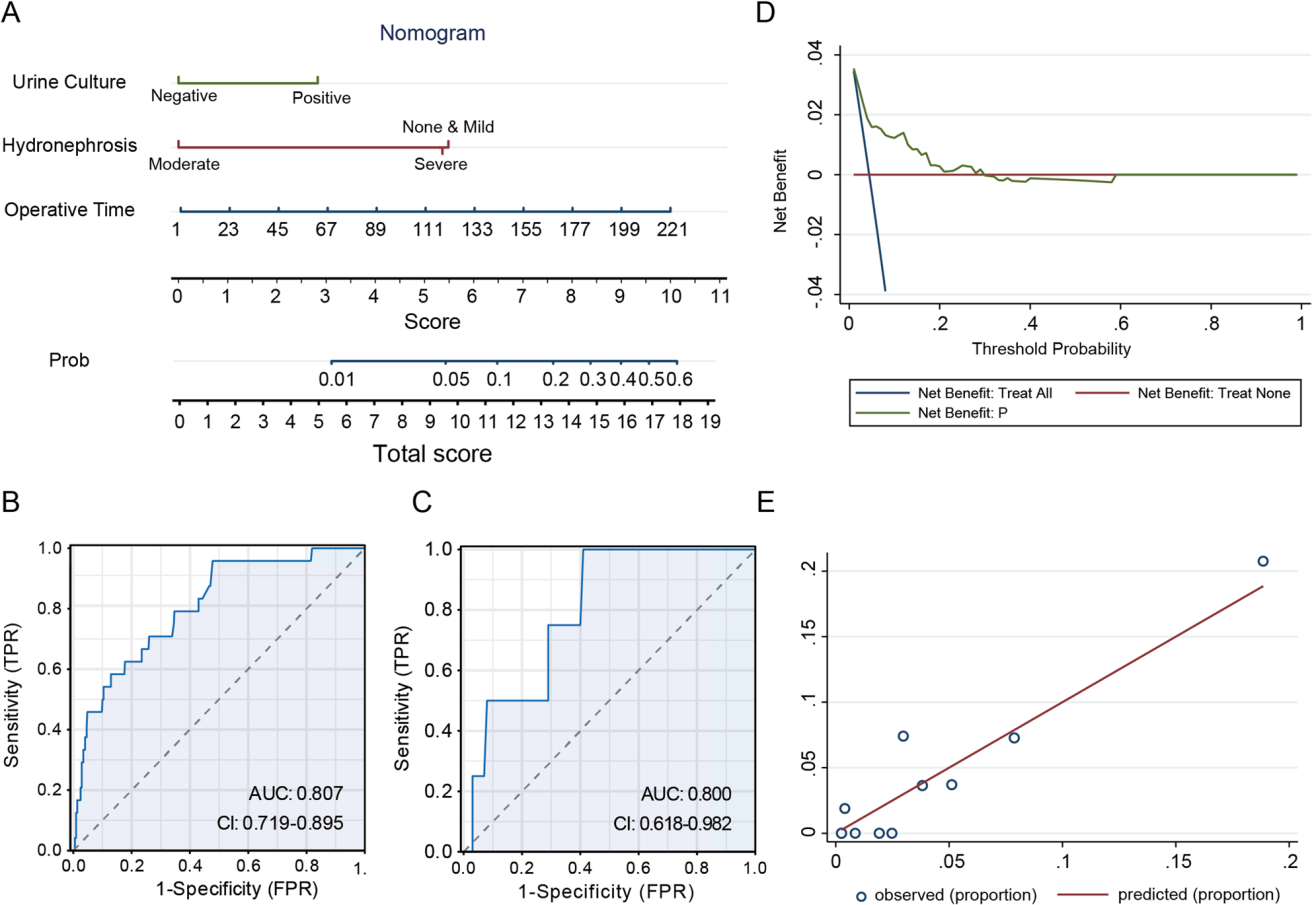
Illustrates the development and validation of a predictive nomogram for severe hemorrhage following PCNL. The nomogram, which incorporates urine culture, hydronephrosis, and operative time, is presented in Fig. 2**A**. The model demonstrated robust predictive performance, achieving an AUC of 0.807 (95% CI: 0.719–0.895) in the training set (Fig. 2**B**) and a consistent AUC of 0.800 (95% CI: 0.618–0.982) in the validation set (Fig. 2**C**). Decision curve analysis (Fig. 2**D**) indicated that the nomogram offered a positive net benefit across a range of threshold probabilities, suggesting its potential clinical applicability. Furthermore, the calibration curve (Fig. 2**E**) revealed excellent agreement between the nomogram's predictions and the actual observed incidence of severe hemorrhage, confirming the model's accuracy and reliability

The hemorrhage prediction model based on these variables also showed good calibration (Hosmer-Lemeshow test, *P* = 0.263) and predictive performance, with an AUC of 0.807 (95% CI: 0.719–0.895) in the development cohort and 0.800 (95% CI: 0.618–0.982) in the validation cohort (Fig. 2).

Subgroup analysis based on the degree of hydronephrosis showed that among the three groups of patients—no/mild hydronephrosis (219 cases), moderate hydronephrosis (227 cases), and severe hydronephrosis (262 cases)—the incidence of severe postoperative hemorrhage was 6.4% (14/219), 0.9% (2/227), and 6.1% (16/262), respectively, while stone clearance rates were 84.9%, 92.1%, and 81.7%, respectively. This indicates that patients with moderate hydronephrosis had the lowest risk of severe postoperative hemorrhage and the highest stone clearance rate.

Notably, among patients who did not develop SIRS (666 patients), the incidence of severe postoperative hemorrhage was 3.6% (24/666); whereas, among patients who developed SIRS (42 patients), this rate increased significantly to 19.0% (8/42), a difference that was statistically significant (*P* < 0.001). Spearman’s correlation analysis showed a weak but significant positive correlation between postoperative SIRS and severe hemorrhage (*r* = 0.176, *P* < 0.001), suggesting that these two complications may not be entirely independent but rather exhibit some association.

## Discussion

Since its introduction, percutaneous nephrolithotomy has become the gold standard for treating kidney stones larger than 2 cm. However, complications associated with PCNL, including infection, hemorrhage, injury to adjacent organs, and urine extravasation, remain major clinical challenges. Among these, complications such as postoperative systemic inflammatory response syndrome and severe hemorrhage directly threaten patient safety and prognosis [14]. Early warning and intervention for the occurrence of such complications can help reduce the risk of adverse postoperative events [15].

If not promptly diagnosed and treated, SIRS can progress to urosepsis or even septic shock. Studies have shown that compared to sepsis from other causes, urosepsis is characterized by acute onset, poor prognosis, and high mortality, highlighting the critical importance of early identification and intervention for SIRS after PCNL [16]. This study excluded patients with pre-existing severe infections. Subsequent statistical analysis identified positive preoperative urine culture, residual stones, and prolonged operative time as independent risk factors for postoperative SIRS. The clinical prediction model constructed based on these three variables demonstrated good fit, with an AUC of 0.761 (95% CI: 0.659–0.863) in the development cohort and 0.773 (95% CI: 0.586–0.961) in the validation cohort, indicating reasonable discriminatory ability.

Clinically, minor bleeding is usually not life-threatening. Therefore, this study defined severe hemorrhage as hemorrhage requiring blood transfusion and/or interventional embolization. Multivariate analysis identified moderate hydronephrosis, positive preoperative urine culture, and prolonged operative time as independent factors influencing postoperative severe hemorrhage. Among these, moderate hydronephrosis was a protective factor, while positive preoperative urine culture and prolonged operative time increased the risk of hemorrhage. The severe hemorrhage prediction model constructed based on these variables also performed well, with an AUC of 0.807 (95% CI: 0.719–0.895) in the development cohort and 0.800 (95% CI: 0.618–0.982) in the validation cohort.

Regarding the relationship between the degree of hydronephrosis and the risk of hemorrhage after PCNL, existing literature reports are inconsistent. Some studies suggest a protective effect of hydronephrosis: For example, a 2023 case-control study showed that absence of hydronephrosis was an independent risk factor for major hemorrhage after PCNL [17]; studies from 2012 also indicated that lack of hydronephrosis was associated with an increased risk of blood transfusion [18]. Other studies have reached opposite conclusions: Studies from 2023 found that the severe hydronephrosis group had a more significant decrease in hemoglobin and a higher transfusion rate [19]; studies from 2019 to 2023 also confirmed that the degree of hydronephrosis was independently associated with hemorrhage [20, 21]. These discrepancies may stem from differences in study design, hydronephrosis grading criteria, and definitions of hemorrhage.

This study found that patients with moderate hydronephrosis had the lowest risk of severe postoperative hemorrhage (0.9%) and the highest stone clearance rate (92.1%), both superior to the no/mild hydronephrosis group (hemorrhage rate: 6.4%, clearance rate: 84.9%) and the severe hydronephrosis group (hemorrhage rate: 6.1%, clearance rate: 81.7%). This might be attributed to the fact that moderate hydronephrosis preserves relatively intact renal morphology while providing a moderately dilated collecting system, facilitating the establishment of the percutaneous renal tract and surgical manipulation. We hypothesize that in cases with no/mild hydronephrosis, limited working space and thicker renal parenchyma may increase the risk of injury. In cases with severe hydronephrosis, excessive thinning and poor elasticity of the renal cortex might affect the natural hemostatic process postoperatively. Additionally, moderate hydronephrosis provides good exposure and an optimal working space for stones, which may also contribute to higher stone clearance rates. However, these explanations remain hypothetical, as this study’s retrospective design did not allow for direct measurement of parameters such as renal parenchymal thickness, intrarenal vascular distribution, or intrarenal pelvic pressure during surgery. Future research should incorporate quantitative analysis of renal parenchyma and vascular structures using preoperative imaging and conduct prospective studies to validate these hypotheses.

Another important finding is that postoperative SIRS and severe hemorrhage may not be entirely independent events. This study showed that the incidence of severe hemorrhage was significantly higher in SIRS-positive patients (19.0%) compared to SIRS-negative patients (3.6%). Spearman’s correlation analysis also suggested a certain positive correlation between the two (*r* = 0.176, *P* < 0.001). On one hand, this might be partially attributed to the limited sample size and relatively low event rates in this study. On the other hand, it may suggest that the two complications share some risk factors (such as positive urine culture, prolonged operative time), warranting further investigation into their potential common pathophysiological mechanisms. Recent studies indicate that significant blood loss can lead to local tissue hypoxia, reduce concentrations of antibiotics and immune cells in the blood, potentially increasing infection risk and triggering SIRS [22, 23]. Simultaneously, surgical trauma and blood transfusion therapy may further suppress the immune response, creating a vicious cycle. Conversely, the release of inflammatory mediators, thrombocytopenia, and coagulation abnormalities associated with SIRS may increase or exacerbate hemorrhage risk. Multiple studies have shown that preoperative urinary tract infection is a risk factor for postoperative hemorrhage, possibly related to inflammatory endothelial vascular injury [24]. Therefore, postoperative SIRS and severe hemorrhage may have pathophysiological interactions, promoting each other, but the specific mechanisms require further elucidation.

Currently, preoperative urine culture is a key method for detecting urinary tract infections and guiding preoperative prophylactic antibiotic use [25]. For patients with positive preoperative urine cultures, antibiotic treatment has been proven effective in reducing the incidence of postoperative complications [26]. In this study, positive preoperative urine culture was identified as a common risk factor for both postoperative SIRS and severe hemorrhage, consistent with findings from many other studies reporting an increased risk of postoperative hemorrhage and infectious complications in such patients [27, 28]. Interestingly, some research indicates that the true causative pathogens for postoperative infections are often those found in stone cultures rather than preoperative urine cultures [29, 30]. However, stones are only obtained intraoperatively, and culture results become available postoperatively, by which time patients may have already developed infection or even sepsis. Prolonged operative time is a recognized independent risk factor for severe complications after PCNL. Potential mechanisms may include: (1) Prolonged wound exposure time, potentially lowering patient immunity and increasing infection risk; (2) Higher likelihood of severe hemorrhage and transfusion requirements, which may weaken the body’s ability to fight infection; (3) Association with the inherent complexity of the stone (e.g., multiple stones, high stone burden); (4) Increased risk of irrigant absorption and mucosal injury, facilitating the entry of bacteria/toxins into the bloodstream, thus predisposing to SIRS and hemorrhage [29]. Traditionally, the presence of residual fragments > 4 mm on postoperative imaging has been defined as clinically significant residual fragments. However, emerging research suggests that achieving a truly stone-free state (potentially defined by a lower threshold, e.g., ≤ 2 mm) is associated with lower rates of postoperative complications and re-intervention [31, 32]. Nevertheless, pursuing smaller residual fragments often requires longer operative time and may incur greater surgical trauma. Whether the benefits outweigh the potential harms requires further investigation.

In the current era emphasizing minimally invasive techniques, procedures with smaller tracts such as Mini-PCNL and Ultra-Mini-PCNL are increasingly popular. Studies suggest that for smaller stones, these methods may achieve comparable stone-free rates while potentially reducing complications [33]. However, as stone diameter and burden increase, the limitations of smaller tracts may lead to decreased stone-free rates and significantly prolonged operative time, thereby increasing the risks of infection and hemorrhage [34].

This study investigated risk factors for severe complications after PCNL and constructed risk prediction models. Positive preoperative urine culture and prolonged operative time were identified as common risk factors for both outcomes. Interestingly, compared to mild and severe hydronephrosis, moderate hydronephrosis was associated with higher stone clearance rates and a lower risk of severe hemorrhage. Notably, we found a significant correlation between the occurrence of postoperative SIRS and severe hemorrhage (*P* < 0.001). However, this study has several limitations. First, it is a single-center retrospective design, subject to selection bias. Second, the incidence of outcome events was relatively low, which may affect model stability. Third, the external validation cohort had a limited sample size (*n* = 104), and the generalizability of the results needs further validation through multicenter studies with larger samples. Fourth, the definition of severe hemorrhage relied on clinical transfusion thresholds, and transfusion decisions may vary between centers and physicians; future studies could use more objective indicators to enhance comparability. Finally, due to the limitations of retrospective data, dynamic changes in hemoglobin levels could not be included; future prospective studies should focus on collecting and analyzing continuous laboratory parameters. Given these limitations, future research should expand the sample size and validate the feasibility of this predictive model using larger, high-quality external datasets.

## Summary

This study successfully identified and validated key independent risk factors for postoperative SIRS and severe hemorrhage after PCNL, and established clinical prediction models with moderate-to-good discriminatory ability, which may serve as auxiliary tools for clinical risk stratification. Moderate hydronephrosis may be a protective factor against severe postoperative hemorrhage. A significant but weak correlation was observed between the occurrence of SIRS and severe hemorrhage, suggesting that the two complications may share some risk factors rather than having a strong direct causal relationship. These factors should be considered together in clinical management. The above findings warrant further validation through multicenter prospective studies.

## Supplementary Information

Supplementary Material 1.

Supplementary Material 2.

## Data Availability

The datasets used and analyzed during the current study are available from the corresponding author on reasonable request.

## Abbreviations

AUC: Area under curve
BMI: Body mass index
CI: Confidence interval
PCNL: Percutaneous nephrolithotomy
OR: Odds ratio
ROC: Receiver operating characteristic curve
SIRS: Systemic inflammatory response syndrome
VIF: Variance Inflation Factor
