# Supplementary material for: Development and validation of predictive models for SIRS and severe hemorrhage following percutaneous nephrolithotomy: the role of hydronephrosis and inter-correlation
Source: BMC Surg. 2026 Apr 11;26:398. doi: 10.1186/s12893-026-03721-6 (PMC13251231; doi:10.1186/s12893-026-03721-6)
Supplement: Supplementary file 1 — Supplementary Material 1. [file 12893_2026_3721_MOESM1_ESM.docx]

| Supplementary Table 1: Baseline Preoperative Characteristics, Stone-related Data, and Surgical Data of the Included 708 Patients | | | | | | | | |  |  |
| --- | --- | --- | --- | --- | --- | --- | --- | --- | --- | --- |
|  | SIRS(-) | | SIRS(+) | | Severe Hemorrhage(-) | | Severe Hemorrhage(+) | | |  |
| Sex,N(%) | |  | |  | |  | |  | | |
| Male | | 432(64.9) | | 25(59.5) | | 437(64.6) | | 20(62.5) | | |
| Female | | 234(35.1) | | 17(40.5) | | 239(49.2) | | 12(37.5) | | |
| Surgical,N(%) | |  | |  | |  | |  | | |
| None | | 545(81.8) | | 37(88.1) | | 556(82.2) | | 26(81.3) | | |
| Within 1 month | | 35(5.3) | |  | | 33(4.9) | | 2(6.30 | | |
| Beyond 1 month | | 86(12.9) | | 5(11.9) | | 87(12.9) | | 4(12.5) | | |
| Preoperative Hb,N(%) | |  | |  | |  | |  | | |
| <120 g/L | | 153(23.1) | | 12(28.6) | | 151(22.5) | | 14(43.8) | | |
| ≥120 g/L | | 509(76.9) | | 30(71.4) | | 521(77.5) | | 18(56.2) | | |
| Hydronephrosis,N(%) | |  | |  | |  | |  | | |
| None & Mild | | 204(30.6) | | 15(35.7) | | 205(30.3) | | 14(43.8) | | |
| Moderate | | 216(32.4) | | 11(26.2) | | 225(33.3) | | 2(6.3) | | |
| Severe | | 246(36.9) | | 16(38.1) | | 246(36.4) | | 16(50) | | |
| BMI,N(%) | |  | |  | |  | |  | | |
| <24 kg/m^2^ | | 332(50.7) | | 26(65) | | 337(50.8) | | 21(65.6) | | |
| ≥24 kg/m^2^ | | 323(49.3) | | 14(35) | | 326(49.2) | | 11(34.4) | | |
| Urine Culture | |  | |  | |  | |  | | |
| Negative | | 451(88.3) | | 20(58.8) | | 464(87.3) | | 17(68) | | |
| Positive | | 60(11.7) | | 14(41.2) | | 66(12.7) | | 8(32) | | |
| Single Stone,N(%) | |  | |  | |  | |  | | |
| Yes | | 493(80.4) | | 30(90.9) | | 498(80.5) | | 25(92.6) | | |
| No | | 120(19.6) | | 3(9.1) | | 121(19.5) | | 2(7.4) | | |
| Residual Stones, N (%) | |  | |  | |  | |  | | |
| No | | 580(87.1) | | 29(69) | | 590(87.3) | | 19(59.4) | | |
| Yes | | 86(12.9) | | 13(31) | | 86(12.7) | | 13(40.6) | | |
| Urine Nitrite, N (%) | |  | |  | |  | |  | | |
| Negative | | 626(96.9) | | 36(87.8) | | 633(96.6) | | 29(90.6) | | |
| Positive | | 20(3.1) | | 5(12.2) | | 22(3.4) | | 3(9.4) | | |
| Urine White Blood Cells, N (%) | |  | |  | |  | |  | | |
| Negative | | 309(47.8) | | 10(24.4) | | 308(47) | | 11(34.4) | | |
| Positive | | 337(52.5) | | 31(75.6) | | 347(53) | | 21(65.6) | | |
| Age | | 51(39，64) | | 49(35，62) | | 51(39,71) | | 55(38,71) | | |
| Stone Diameter | | 1.8(1.4,2.3) | | 2(1.6,3) | | 1.8(1.4,2.3) | | 1.7(1.3,2.4) | | |
| Blood Glucose | | 5.06(4.72,5.48) | | 4.9(4.68,5.38) | | 5.04(4.71,5.46) | | 5.19(4.82,5.68) | | |
| Operative Time | | 69(52,90) | | 98(72,128) | | 69(52,90) | | 103(68,137) | | |
| Prothrombin Time | | 13.2(12.8,13.6) | | 13.2(12.7,13.5) | | 13.2(12.8,13.6) | | 13.1(12.8,13.7) | | |
| Albumin | | 40.5(38.4,42.7) | | 40.1(38.4,42.6) | | 40.5(38.5,42.7) | | 39.7(37.1,42.9) | | |
| Globulin | | 28.3(25.4,32.4) | | 30.1(26.3,34) | | 28.3(25.4,31.4) | | 29.9(26.2,33.5) | | |
| Triglycerides | | 1.33(0.98,1.96) | | 1.36(1.02,2.04) | | 1.34(0.99,1.96) | | 1.05(0.91,2.22) | | |
| Cholesterol | | 4.02(3.49,4.63) | | 4.03(3.61,4.54) | | 4.02(3.49,4.61) | | 4.03(3.48,4.95) | | |
| Serum Creatinine | | 85(70,104) | | 88(75,133) | | 85(70,104) | | 92(76,137) | | |
| C-reactive Protein | | 1.6(0.6,5.4) | | 4.1(0.8,12.9) | | 1.7(0.6,5.4) | | 3.5(0.7,21) | | |
| Albumin-to-Globulin Ratio | | 1.45(1.16,1.74) | | 1.36(1.11,1.6) | | 1.45(1.16,1.73) | | 1.37(1.03,1.68) | | |

Note: All units are reported in the International System of Units. Operative time is recorded in minutes (min), preoperative prothrombin time in seconds (s), and stone diameter in centimeters (cm).
