# Supplementary material for: Development and validation of predictive models for SIRS and severe hemorrhage following percutaneous nephrolithotomy: the role of hydronephrosis and inter-correlation
Source: BMC Surg. 2026 Apr 11;26:398. doi: 10.1186/s12893-026-03721-6 (PMC13251231; doi:10.1186/s12893-026-03721-6)
Supplement: Supplementary file 2 — Supplementary Material 2. [file 12893_2026_3721_MOESM2_ESM.docx]

| Supplementary Table 2: Baseline Characteristics of Patients in the External Validation Cohort of the Prediction Model | | | |
| --- | --- | --- | --- |
| Total (N = 104) | | | |
| Postoperative SIRS, N(%) | | Residual Stones, N (%) |  |
| SIRS(+) | 8(7.7) | No | 75(72.1) |
| SIRS(-) | 96(92.3) | Yes | 29(27.9) |
| Severe Hemorrhage, N(%) | | Hydronephrosis,N(%) |  |
| Severe Hemorrhage (+) | 4(3.8) | None & Mild | 28(26.9) |
| Severe Hemorrhage (-) | 100(96.2) | Moderate | 49(47.1) |
| Urine Culture, N(%) |  | Severe | 27(26) |
| Positive | 20(19.2) | Operative Time | 68(52,97) |
| Negative | 84(80.8) |  |  |
